# LCAMNet: a lightweight model for apple leaf disease classification in natural environments

**DOI:** 10.3389/fpls.2025.1626569

**Published:** 2025-08-12

**Authors:** Yuanyuan Jiao, Honghui Li, Xueliang Fu, Buyu Wang, Kaiwen Hu, Shuncheng Zhou, Daoqi Han

**Affiliations:** ^1^ College of Computer and Information Engineering, Inner Mongolia Agricultural University, Hohhot, China; ^2^ Key Laboratory of Smart Animal Husbandry at Universities of Inner Mongolia Autonomous Region, Hohhot, China

**Keywords:** apple leaf disease, image classification, deep learning, triplet attention mechanism, FGVC8 dataset

## Abstract

Apple leaf diseases severely affect the quality and yield of apples, and accurate classification is crucial for reducing losses. However, in natural environments, the similarity between backgrounds and lesion areas makes it difficult for existing models to balance lightweight design and high accuracy, limiting their practical applications. In order to resolve the aforementioned problem, this paper introduces a lightweight converged attention multi-branch network named LCAMNet. The network integrates depthwise separable convolutions and structural re-parameterization techniques to achieve efficient modeling. To avoid feature loss caused by single downsampling operations, a dual-branch downsampling module is designed. A multi-scale structure is introduced to enhance lesion feature diversity representation. An improved triplet attention mechanism is utilized to better capture deep lesion features. Furthermore, a dataset named SCEBD is constructed, containing multiple common disease types and interference factors under natural environments, realistically reflecting orchard conditions. Experimental results show that LCAMNet achieves 92.60% accuracy on the SCEBD and 95.31% on a public dataset, with only 0.03 GFLOPs and 1.30M parameters. The model maintains high accuracy while remaining lightweight, enabling effective apple leaf disease classification in natural environments on devices with limited resources.

## 1 Introduction

Apple (Malus domestica), a member of the Rosaceae family, is one of the most widely cultivated and commonly consumed fruits worldwide. China is the largest apple producer globally, accounting for 58.3% of the world’s total apple production in 2022, ranking first in the world (Association, 2023). However, the growth of apple leaves is frequently threatened by pathogens such as fungi and viruses, which can lead to various diseases and result in significant economic losses (Ali et al., 2024). Therefore, timely detection and accurate identification of apple leaf diseases are of great importance.

Traditional apple leaf disease detection methods primarily rely on expert visual inspection and experience (Sheng et al., 2022), which are time-consuming, labor-intensive, and highly susceptible to subjective factors such as fatigue, expertise level, and environmental variability (Zhang et al., 2024). To improve efficiency, researchers have proposed machine learning-based approaches (Predić et al., 2022a), which extract handcrafted global features—such as color, texture, and shape—and use traditional image processing techniques combined with classifiers for disease recognition (Bacanin et al., 2022; Bukumira et al., 2022). However, these methods have notable limitations: (1) Handcrafted features often lack the descriptive power to capture local characteristics of complex lesions accurately; (2) They are sensitive to noise, lighting variations, and background clutter, resulting in unstable features and reduced classification accuracy.

With the rise of deep learning, convolutional neural networks (CNNs) have demonstrated strong performance in crop disease classification tasks (Wang et al., 2024; Petrovic et al., 2024; Huang et al., 2025). CNNs can automatically learn discriminative features from raw images, eliminating the need for manual feature engineering. However, existing CNN-based models still face three major challenges in apple leaf disease recognition: (1) Most models are trained on images captured in controlled laboratory environments, lacking high-quality samples collected under real-world field conditions, which limits generalization and practical deployment; (2) Many high-accuracy models are architecturally complex and have large numbers of parameters, making them difficult to deploy on resource-constrained mobile or edge devices. While model compression techniques such as pruning can partially reduce computational demands, it remains challenging to balance accuracy and efficiency (Predić et al., 2022b).

To address these issues, this study constructs a real-field apple leaf disease image dataset. Based on this dataset, we propose an efficient and lightweight deep neural network, named LCAMNet. The network integrates depthwise separable convolution and structural re-parameterization techniques to achieve lightweight yet effective modeling. In addition, it incorporates multi-scale downsampling and multi-scale feature extraction modules to enhance the representation of diverse lesion characteristics. An improved triplet attention mechanism is also introduced to strengthen the modeling of deep lesion features. Experimental results demonstrate that LCAMNet achieves an accuracy of 92.60% on the SCEBD dataset and 95.31% on a public dataset, while requiring only 0.03 GFLOPs and 1.30 million parameters, making it highly suitable for deployment in resource-limited environments for apple leaf disease classification.

The main contributions of this paper are as follows:

1. A dual-branch downsampling module is designed. Applying different downsampling operations to channels and using channel shuffle to improve feature fusion between channels. This avoids information loss caused by single downsampling strategies and improves recognition accuracy.
2. A multi-scale feature extraction module is proposed. Four feature extractors are designed to capture diverse features of the lesion regions from different receptive fields. In addition, channel separation is used to reduce the convolutional computation cost, and the channel shuffling method solves the information isolation issue caused by grouped convolutions, promoting feature fusion across different groups of channels.
3. An improved triplet attention mechanism is introduced. The original 7x7 convolution is replaced by two cascaded 3x3 convolutions, which not only enhance the deep lesion feature modeling ability but also effectively reduce the model parameter size.
4. A novel dataset, SCEBD, is developed by aggregating images from four distinct sources and employing a variety of data augmentation techniques. It serves to enable a comprehensive evaluation of LCAMNet and significantly enhances its generalization capability.

The structure of this paper is as follows: Section 2 reviews related work. Section 3 presents the dataset and the proposed model. Section 4 outlines the experimental setup and results. Section 5 provides the conclusion.

## 2 Related work

Since extracting effective features from crop disease images is a critical and challenging task, and deep learning techniques have the capability to automatically learn features from raw images, research in this field primarily focus on designing high-performance model architectures to improve recognition accuracy (Liu et al., 2022b; Liang and Jiang, 2023; Li et al., 2024).

(Tang et al., 2024) improve the Inception module based on ResNet50 and integrate the ResNeXt inverted bottleneck module. Their model is capable of identifying seven categories of apple leaves. (Sun et al., 2025) develop the EMA-DeiT model based on the DeiT, achieving 99.6% accuracy on the PlantVillage dataset for classifying 10 types of tomato diseases and 98.2% accuracy on a dataset containing 6 disease types. (Zhang et al., 2023) introduce a Dilated Inception module into AlexNet, replacing the fully connected layer with global pooling, which effectively recognizes apple leaf diseases under small sample conditions. (Jiang et al., 2023) enhance the feature extraction ability for leaf diseases by integrating channel and spatial attention mechanisms to ResNet18, achieving 98.25% classification accuracy on a 5-class apple leaf disease dataset. Although these studies show good performance in terms of classification accuracy, most models have complex architectures and large numbers of parameters, which limit their deployment in real agricultural scenarios. Consequently, research has shifted toward lightweight designs.

(Cui et al., 2025) combine CNN and Transformer architectures, achieving high accuracy with fewer parameters. (Li et al., 2023) use a multi-branch structure to capture diversified features and apply residual connections between layers to ensure maximum information transfer, maintaining fewer parameters while ensuring good generalization. (Dong et al., 2024) introduce the ECA module into EfficientNetB0 model and apply knowledge distillation to further optimize the model, increasing accuracy without expanding model size. (Ullah et al., 2024) combine convolutional and ViT blocks to capture both local and global features, achieving 96.38% classification accuracy on the FGVC8 dataset. While these models have made significant progress in lightweight design, they still face challenges such as simple experimental datasets, which limit their adoption in real-world agricultural environments. Some studies have begun to focus on more challenging datasets.

(Wang and Cui, 2024) enhance the representational capacity of the model by modifying the convolutional kernels of ShuffleNetV2 and introducing spatial attention and Ghost modules. They also construct a dataset comprising five categories of apple leaf disease images. Experimental results show that the improved model outperforms the baseline model across multiple metrics, with the model parameters totaling only 9.8 MB. (Liu et al., 2022a) build the ALS module based on ShuffleNetV2 using depthwise separable convolutions and channel shuffling, which reduces the computational cost and number of parameters. Furthermore, a knowledge distillation strategy is employed to train the model, further improving its accuracy. This approach enables real-time, automated monitoring of apple leaf pests and diseases on mobile devices. (Liu et al., 2023) also propose a method based on MobileNetV3, in which model parameters are progressively optimized using a univariate approach. A flooding technique is introduced as a novel training strategy to prevent excessive loss minimization. This method achieves superior results on both custom and public datasets. (Li et al., 2025) develop a corn leaf disease recognition model based on MobileNetV3-Large, incorporating a high-frequency feature extraction (HFFE) module to integrate high-frequency image information at the network’s output stage. Additionally, the ACON-C activation function is introduced to enhance the model’s nonlinear representation capacity. Experimental results indicate a 2.1% improvement in average recognition accuracy compared to the baseline model. (Zheng et al., 2023) propose a network architecture optimized for both training and inference. By employing depthwise separable convolutions and structural re-parameterization techniques, along with embedding a parallel dilated attention module, the model achieves the fastest inference speed on a CPU.

Current studies have thoroughly validated the effectiveness of deep learning techniques in plant leaf disease classification, particularly highlighting their potential for application in natural environments. However, existing apple leaf disease datasets still fall short in fully capturing the diversity and complexity of real orchard conditions. Moreover, the increasing complexity of models designed to improve classification accuracy poses challenges for deployment on resource-constrained devices. Therefore, this study focuses on the construction of datasets collected under natural environmental conditions and the design of lightweight network architectures, aiming to achieve efficient model deployment while maintaining high classification accuracy, thus contributing to the development needs of smart agriculture.

## 3 Materials and methods

### 3.1 Image acquisition and preprocessing

This study conducts experiments on two datasets: a public dataset and a self-constructed dataset with natural environmental backgrounds. The specifics of these datasets are detailed in Sections 3.1.1 and 3.1.2, respectively, while the data preprocessing process is explained in Section 3.1.3.

#### 3.1.1 Public dataset FGVC8

The public dataset used in this study is from the CVPR 2021 FGVC8 plant pathology recognition challenge (Thapa et al., 2021). It consists of 18,632 field-captured apple leaf images. The images are taken at various apple maturation stages and during different times of the day, with non-uniform backgrounds. Most of the images have a resolution of 2676x4000. The dataset includes apple leaf images with various disease categories, including alternaria leaf spot, healthy, powdery mildew, rust, and scab. The selected sample sizes for each category are 489, 529, 485, 503, and 504 images, respectively. After preprocessing, these images are used to form the FGVC8 dataset, with the category distribution shown in **Table 1**.

**Table 1:** FGVC8 dataset class distribution.

Class number	Image type	Original image
0	Alternaria leaf spot	489
1	Healthy	529
2	Powdery mildew	485
3	Rust	503
4	Scab	504

#### 3.1.2 Self-constructed natural environmental background dataset

**Figure 1:**
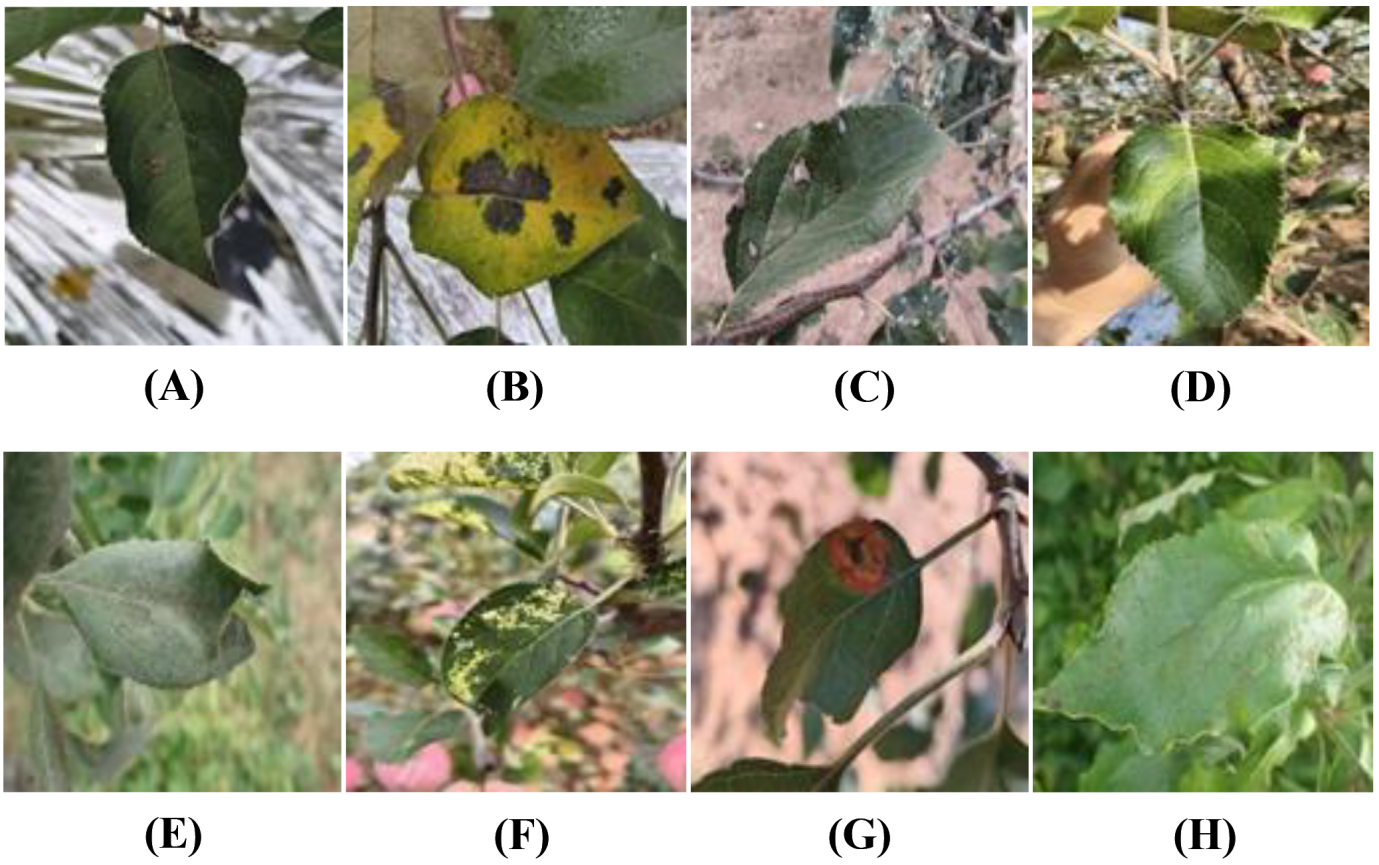
Examples of apple leaf disease images: **(A)** Alternaria leaf spot, **(B)** Brown spot, **(C)** Gray spot, **(D)** Health, **(E)** Powdery mildew, **(F)** Mosaic, **(G)** Rust, **(H)** Scab.

This study also creates a dataset of apple leaf diseases set against a natural environmental background, called SCEBD. The dataset is compiled from four data sources: the FGVC8 dataset, Appleleaf9 (Yang et al., 2022), ATLDSD (Feng and Chao, 2022) and self-collected apple leaf disease images. Some images of alternaria leaf spot, healthy, rust, powdery mildew, and scab are from the FGVC8 dataset, with the following sample sizes: 293, 183, 154, 485, and 484 images, respectively. Images of mosaic are sourced from the Appleleaf9 dataset (a total of 105 images), which combines data from four different apple disease datasets, with varying pixel sizes. Images of gray spot are taken from the ATLDSD dataset (a total of 121 images), captured by a Glory V10 smartphone, with images taken from a real orchard, and the pixel size is 256x256. In addition, images of apple leaf diseases, including alternaria leaf spot, brown spot, gray spot, healthy, mosaic, and rust, are collected from real orchards in Yongning Town, Wafangdian City, Liaoning Province, China, using a smartphone (iQOONeo9Pro). The number of images for each disease is as follows: 192 for alternaria leaf spot, 480 for brown spot, 362 for gray spot, 302 for healthy, 376 for mosaic, and 328 for rust. These images are collected in natural environmental settings that include tree leaves, weeds, and the image resolution is not uniform. The distribution of image types and quantities in the SCEBD is shown in **Table 2**, and examples of apple leaf disease images are shown in **Figure 1**.

**Table 2:** SCEBD class distribution.

Class number	Image type	FGVC8	Private Data	Appleleaf9	ATLDSD	Total images
0	Alternaria leaf spot	293	192	0	0	485
1	Brown spot	0	480	0	0	480
2	Gray spot	0	362	0	121	483
3	Healthy	183	302	0	0	485
4	Powdery mildew	485	0	0	0	485
5	Mosaic	0	376	105	0	481
6	Rust	154	328	0	0	482
7	Scab	484	0	0	0	484

#### 3.1.3 Data preprocessing

**Figure 2:**
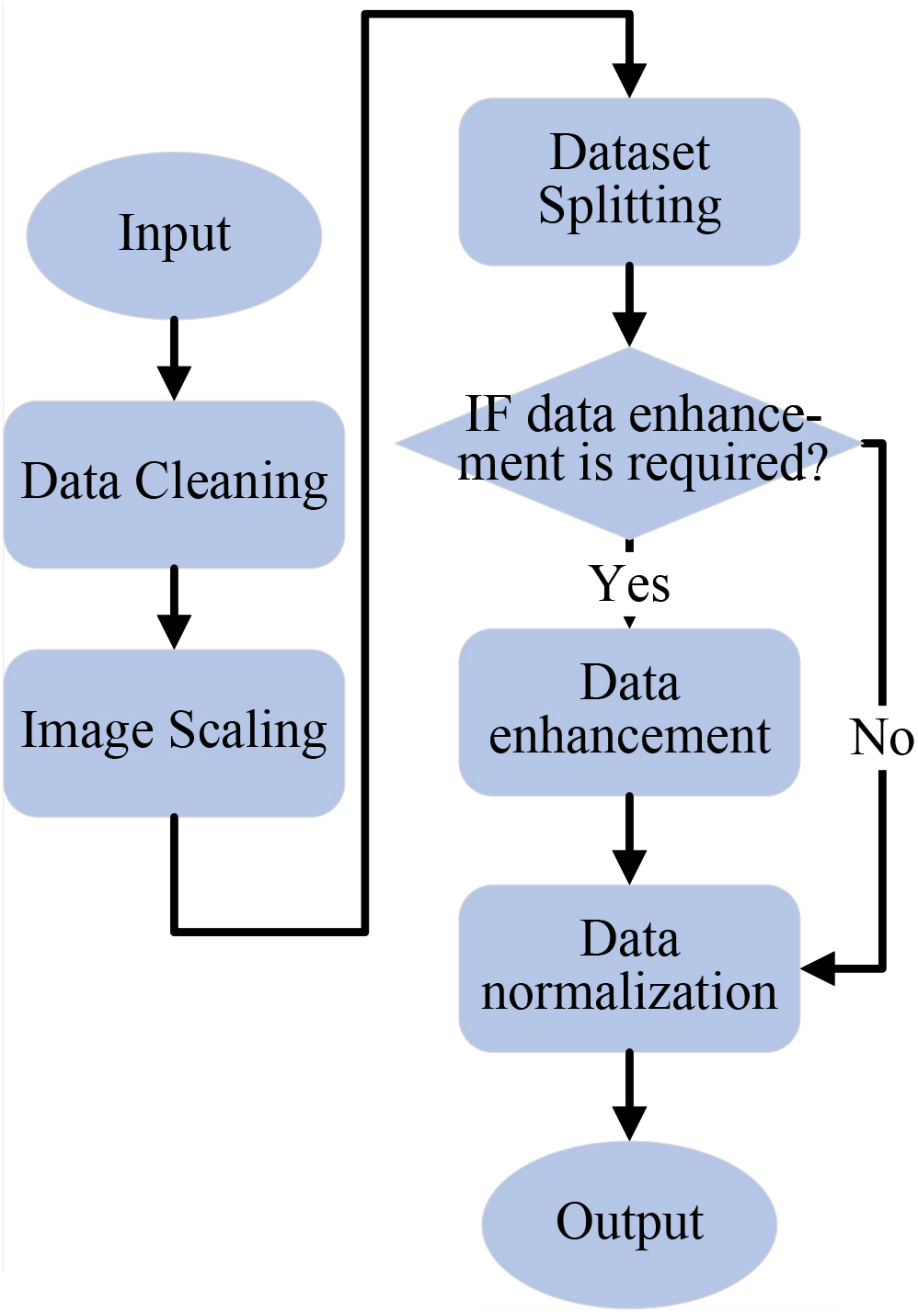
Data preprocessing flowchart.

The collected image data is first cleaned to satisfy criteria, then resized to 224×224 pixels for easier computation. The dataset is split into training, validation, and test sets at a 7:2:1 ratio. Data augmentation is performed only on the training set, while validation and test sets remain unchanged. Next, normalization is performed using the mean and standard deviation for the RGB channels. The data preprocessing flowchart is shown in **Figure 2**.

**Figure 3:**
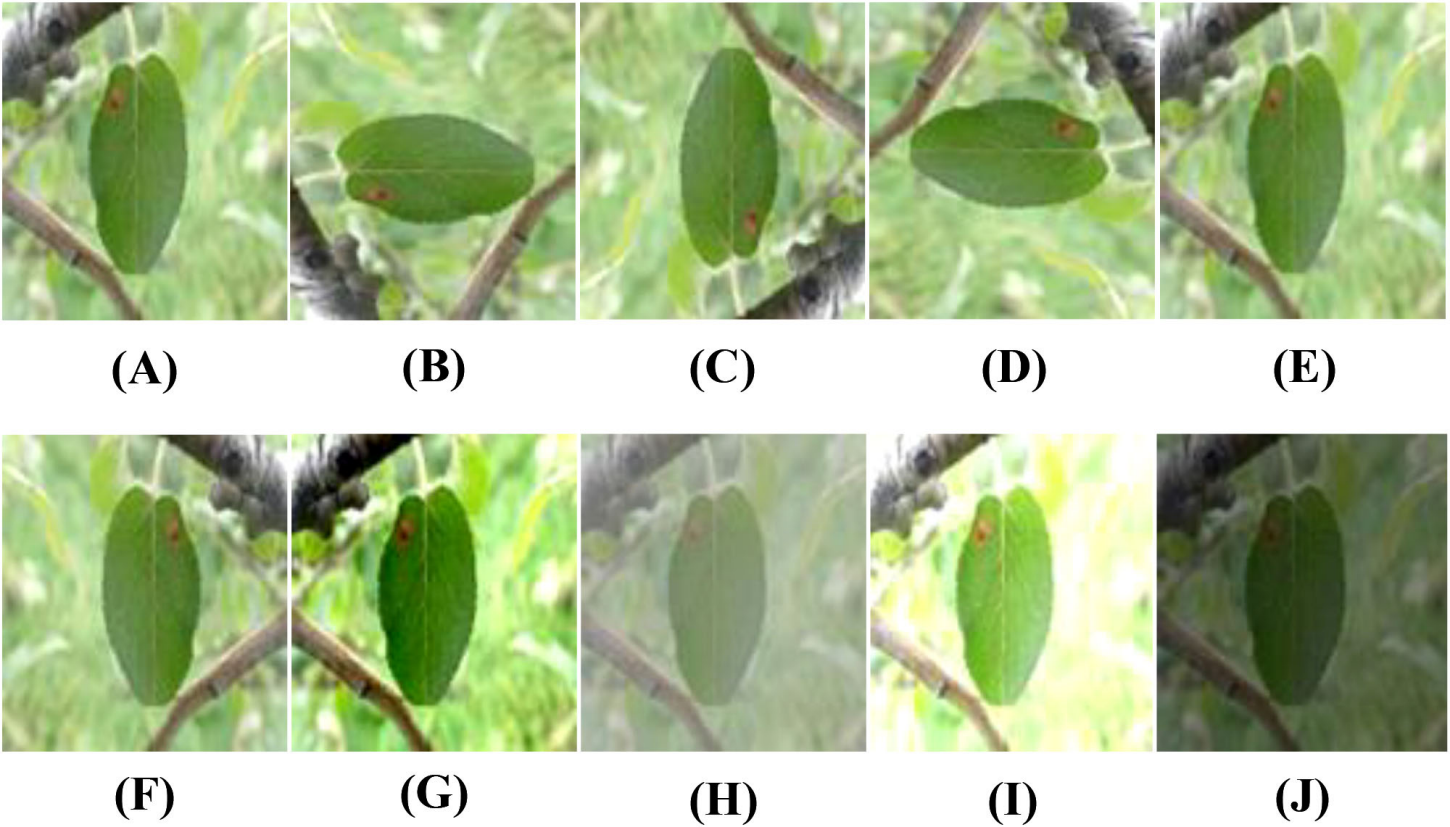
Examples of data augmentation for apple leaf disease images: **(A)** Original, **(B)** Rotated 90, **(C)** Rotated 180, **(D)** Rotated 270, **(E)** Blurred, **(F)** Horizontal flip, **(G)** Contrast high, **(H)** Contrast low, **(I)** Brightness high, **(J)** Brightness low.

To improve the model’s generalization and minimize noise interference, nine types of data augmentation are used on the training images. These include rotations (90°, 180°, and 270°), gaussian blur, random flips (50% probability for both horizontal and vertical flips), contrast enhancement and reduction, and brightness enhancement and reduction. These augmentations increase the number of training images to 10 times the original size. No data augmentation is applied to the test and validation sets. Examples of the augmented images are shown in **Figure 3**.

To prevent instability in model training caused by excessively large or small pixel values and to reduce the risk of overfitting, the images are normalized. Specifically, the mean values for the red, green, and blue channels are set to [0.485, 0.456, 0.406], and the standard deviations are [0.229, 0.224, 0.225]. This normalization method helps accelerate model convergence, improves training stability, and enables more efficient learning of image features.

### 3.2 LCAMNet model

#### 3.2.1 Model structure

**Figure 4:**
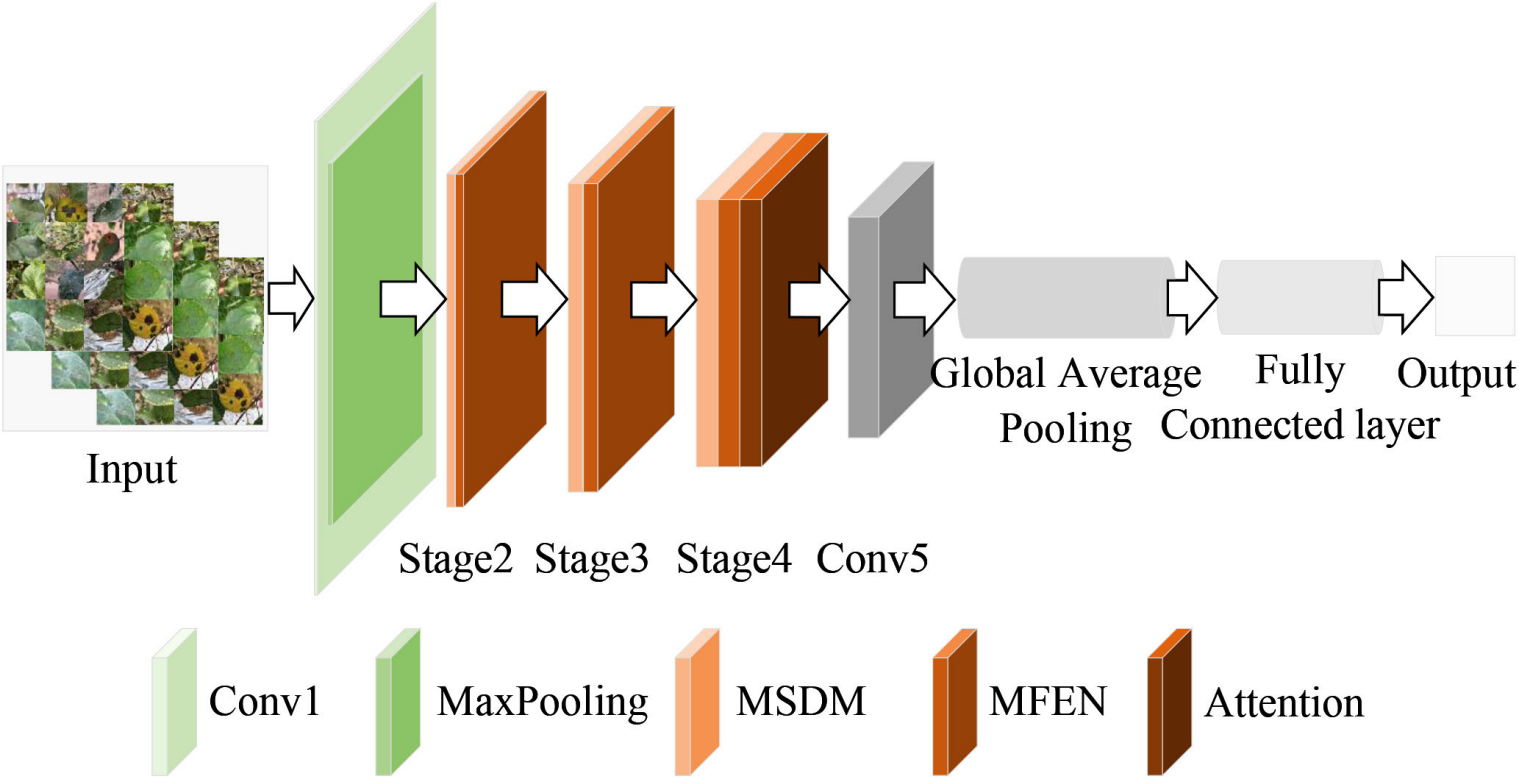
Architecture diagram of LCAMNet.

To address the problem of apple leaf disease classification in natural environments, this paper proposes a lightweight converged attention multi-branch network (LCAMNet). The model includes a 3×3 standard convolutional layer (Conv1), a max pooling layer (MaxPooling), three stage modules (Stage2 to Stage4), a 1×1 convolutional layer (Conv5), a global pooling layer (Global Pooling), and a fully connected layer (FC). The overall structure of the model is shown in **Figure 4**.

LCAMNet initially processes the input image through Conv1 to extract basic features. It then uses MaxPooling for downsampling, reducing the dimensionality of the output features. The subsequent stages, Stage2 to Stage4, contain several key modules, each consisting of a dual-branch downsampling module (DBDM) and a multi-scale feature extraction module (MFEM). The DBDM module performs downsampling on the extracted features, improving computational efficiency while retaining key features. On top of this, the MFEM module captures multi-scale features related to the disease. Both the DBDM and MFEM modules are stacked three times, with each stack extracting deeper features from the previous layer. At the end of Stage4, LCAMNet introduces an improved triplet attention mechanism to further extract crucial feature information. The model then connects a convolutional layer for feature fusion, followed by a global pooling layer and a fully connected layer for classification, outputting the final class results.

#### 3.2.2 Dual-branch downsampling module

Downsampling is often used in convolutional neural networks (CNNs) to reduce the spatial size of feature maps. Pooling is one of the most commonly used downsampling methods, which aggregates pixel values in a local region to decrease the spatial dimensions of feature map, thereby improving model’s robustness to translation variations. Unlikepooling, convolution operations learn convolutional kernel parameters to extract image features, retaining more useful information during the downsampling process and adapting better to various tasks (Li et al., 2023).

**Figure 5:**
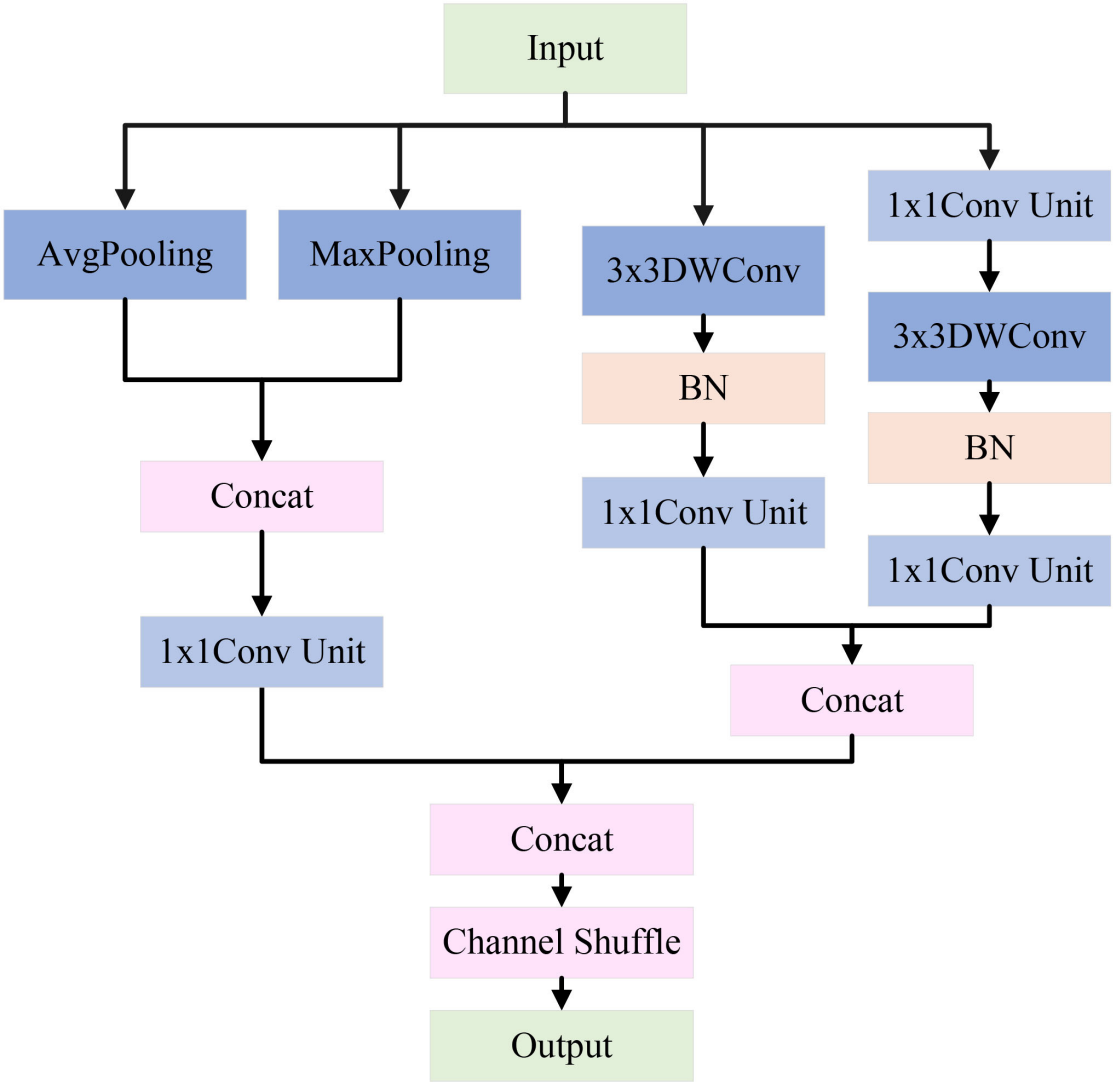
Architecture of DBDM.

However, in natural environmental backgrounds, the classification performance of apple leaf disease images is often weak. A single downsampling strategy may cause the loss of important image features, thus affecting the classification results. To address this challenge, LCAMNet incorporates a dual-branch downsampling module, whose structure is shown in **Figure 5**. This module performs downsampling on the input feature map through both a pooling branch and a convolutional branch. It then concatenates the output features and applies a channel shuffling operation to achieve feature fusion, producing the final output features. The specific design of the two branches is described in the following paragraphs.

**Figure 6:**
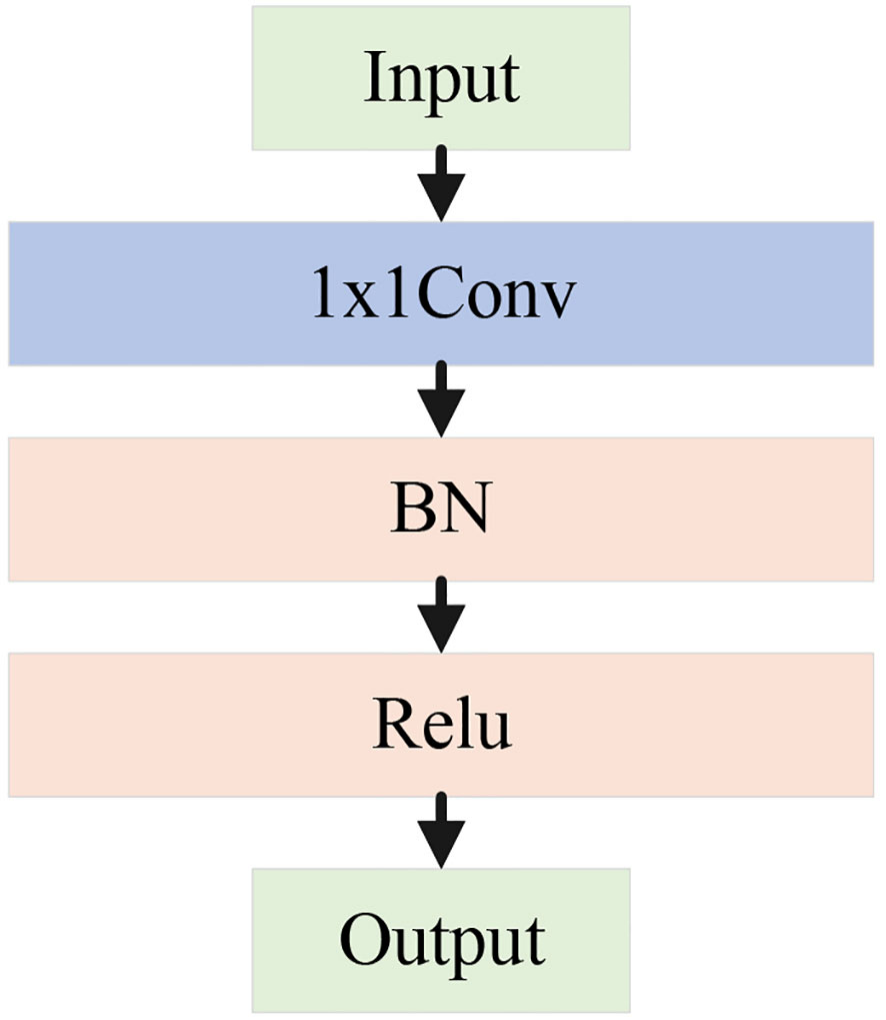
Architecture diagram of the 1×1 conv unit.

The pooling branch applies max pooling and average pooling to extract global and salient features from apple leaf disease images. Specifically, average pooling averages the pixels within pooling region, effectively suppressing local noise and enabling stable feature extraction of diseased areas under complex background interference. In contrast, max pooling selects the maximum value from the pooling window to extract salient features, enhancing the most representative texture and morphological changes in the diseased area. This is crucial for distinguishing apple leaf lesions from the salient regions in the complex background. By concatenating the results of max pooling and average pooling, the module achieves a complementary combination of global and salient features. Subsequently, a 1×1 convolution unit is used to aggregate channels, which includes a 1×1 convolution, batch normalization (BN), and a ReLU activation function. This step enhances model’s ability to capture nonlinear feature representations, as shown in **Figure 6**.

The convolutional branch consists of convolutional downsampling modules, which aim to reduce dimensions while effectively extracting feature information. Specifically, a 3×3 depthwise convolution with a stride of 2 is used to extract local features, halving the spatial resolution. The parallel convolutional branches double the number of channels via channel concatenation to maintain the same channel dimension as the pooling branch. By concatenating the features extracted from both branches and applying channel shuffling, the model enhances the interaction between channels, effectively preserving the key features of the apple leaf disease areas. The pseudocode for this module is shown in **Algorithm 1**.

Algorithm 1 **Downsampling Process of DBDM.**

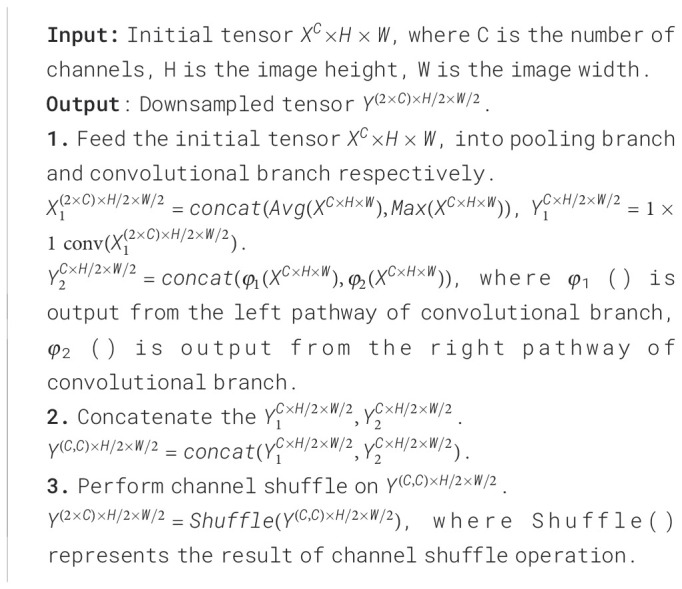

#### 3.2.3 Multi-Scale Feature Extraction Module

**Figure 7:**
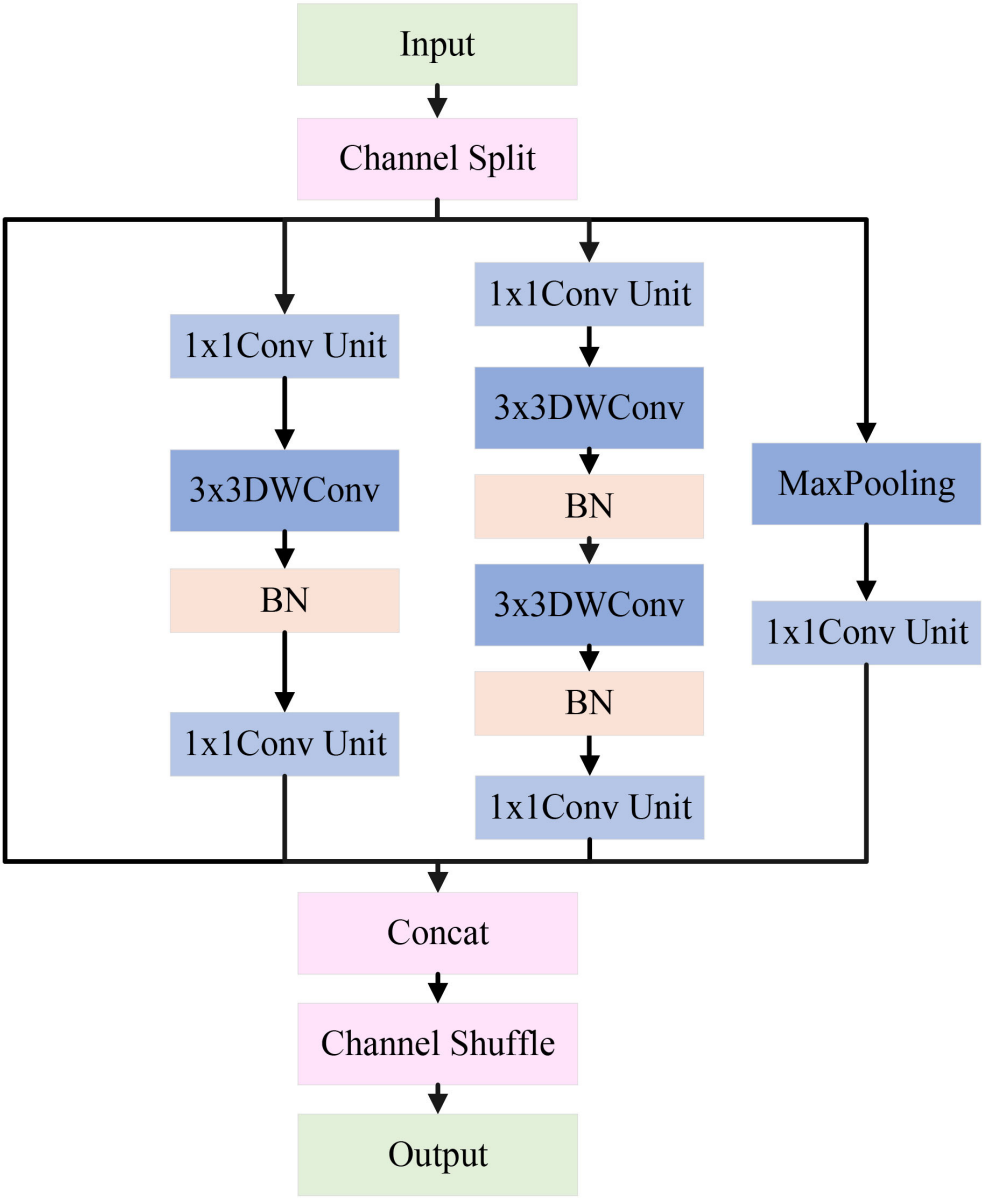
Architecture of MFEM.

Apple leaf disease classification is often weak in complex environments, a single-size convolutional kernel may not effectively extract image features. To address this issue, this study constructs a Multi-Scale Feature Extraction Module (MFEM). Its architecture is shown in **Figure 7**.

The input features first go through a channel separation operation, evenly dividing the channels into four independent branches: the feature-preserving branch, the local detail branch, the deep feature branch, and the salient feature branch. These branches are arranged from left to right, each extracting features at different scales of apple leaf disease image. The feature maps from each branch are fused through concatenation and channel shuffle to improve channel interaction. The detailed design of each branch is explained below.

In the feature-preserving branch, input features are directly forwarded via a skip connection to produce output. This helps distinguish low-level information and mitigates the degradation problem of deep networks. This branch is crucial for capturing subtle local variations in the image features. For example, in apple leaf disease classification, it helps distinguish minor differences between background noise (such as lighting changes, weeds, or shadows) and disease areas (such as brown spots or leaf discoloration).

In the local detail branch, the input feature map first goes through a 1×1 conv unit to adjust the number of channels. Then, the feature map is passed through a 3×3 depthwise convolution and BN. The depthwise convolution performs independently on each input channel, enabling the extraction of fine-grained features within each channel. Finally, a 1×1 conv unit is applied to fuse the features extracted by the depthwise convolution from each channel. This branch mainly extracts features related to the edges of lesions, local texture changes, and small disease regions in apple leaf disease images.

In the deep feature branch, input feature map first goes through a 1×1 conv unit, followed by two 3×3 depthwise convolutions and BN. Afterward, another 1×1 conv unit is applied. The cascading 3×3 convolution layers form a deeper feature extraction unit, capable of capturing more complex local patterns and multi-layered details, such as lesions of varying sizes and morphological changes in apple leaf disease images.

In the salient feature branch, the input feature map passes through a max-pooling layer to extract features, followed by a 1×1 conv unit to adjust the number of channels. In natural settings, max pooling helps enhance the network’s ability to detect salient disease regions in apple leaf images, especially when the contrast between the background and disease features is low, making it more effective in highlighting key features.

At last, the feature maps from all four branches are concatenated to merge multi-scale features, constructing a richer global representation. Additionally, channel shuffling is introduced to promote cross-branch feature exchange and reorganization, thereby enhancing the module’s ability to represent features of the apple leaf disease regions. The pseudocode for this module is shown in **Algorithm 2**.

Algorithm 2 **The feature extraction process of MFEN.**

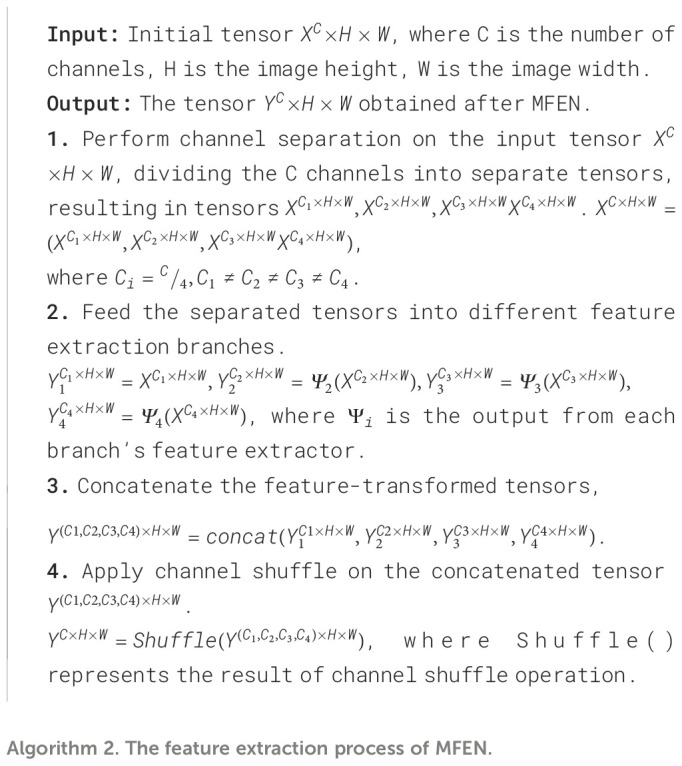

#### 3.2.4 Improved triplet attention mechanism

In recent years, attention mechanisms demonstrate significant advantages in computer vision by helping the model focus on important regions, thus enhancing classification accuracy. Although this study introduces multi-scale branches in the feature extraction layer to capture rich features, challenges remain in capturing deeper features. To solve this, this paper introduces an attention mechanism that captures information from different dimensions, improving the model’s ability to detect apple leaf disease regions.

**Figure 8:**
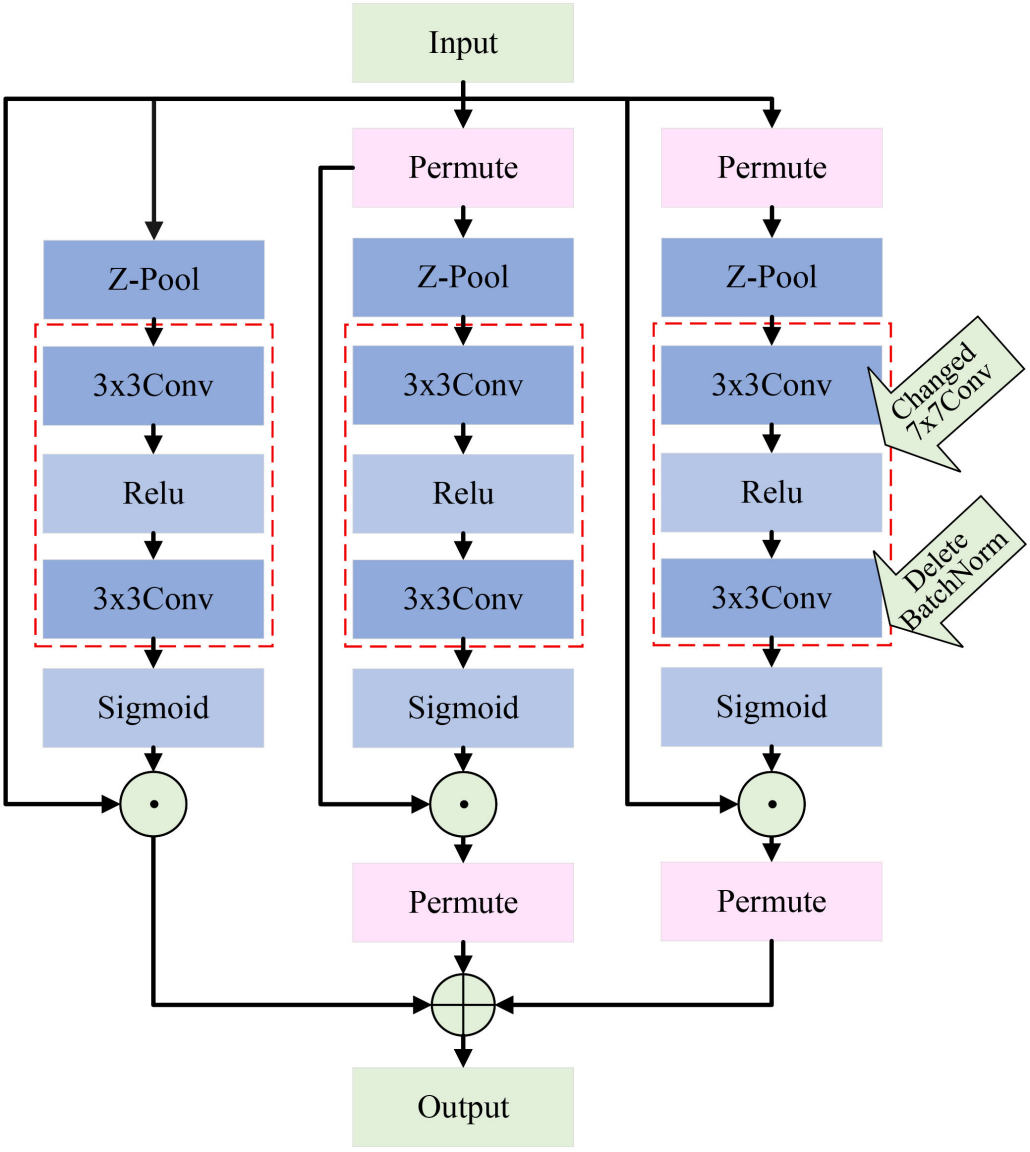
Architecture of the improved triplet attention mechanism.

The triplet attention mechanism (Misra et al., 2021) captures interactions between channels and spatial dimensions through two branches. A third branch is used to create spatial attention, and the outputs from all three branches are combined to form the final attention features. This paper improves upon the original triplet attention mechanism. Specifically, we replace the original 7×7 convolution used for feature extraction with two cascading 3×3 convolutions. This modification allows us to maintain sufficient feature extraction capability without increasing the computational burden. Additionally, we introduce the ReLU activation function (Nair and Hinton, 2010) between the two 3×3 convolutions, which effectively controls the network’s sparsity and enhances its ability for nonlinear transformations. It is worth noting that we remove the original BN module. Experimental analysis reveals that the BN module does not have a significant effect and, in fact, increases computational load. Through these improvements, we significantly enhance the model’s efficiency and practical application performance while ensuring effective cross-dimensional feature modeling. The structure of the improved mechanism is shown in **Figure 8**.

## 4 Experimental analysis

### 4.1 Experimental environment

The experimental hardware in this study uses an Intel(R) Core(TM) i7–6700 CPU @ 3.40GHz processor, and the operating system is Windows 10. Model training and testing are accelerated using a GPU, specifically a Tesla V100S-PCIE-32GB graphics card. The software environment includes Python 3.8.19, the PyTorch 2.4.1 framework, and the CUDA toolkit 12.4.

The number of iterations is set to Epoch=60, with a batch size of 32. The model training uses the Stochastic Gradient Descent (SGD) algorithm, which is one of the most commonly used optimization methods in machine learning. The initial learning rate for SGD is set to 0.0001. A cosine annealing schedule is applied for the first 50 epochs, gradually decreasing the learning rate from 1e-4 to 1e-6, and the learning rate is kept constant at 1e-6 from epoch 50 to 60.

### 4.2 Evaluation criterion

In this study, six evaluation metrics are employed to assess the performance of the proposed model: Accuracy, Precision, Recall, F1-Score, Kappa, and Matthews Correlation Coefficient (MCC). Their definitions are as follows: Accuracy refers to the ratio of correctly predicted samples to the total number of samples. Precision is the proportion of correctly predicted positive samples among all samples predicted as positive. Recall represents the proportion of correctly predicted positive samples among all actual positive samples. F1-Score is the harmonic mean of Precision and Recall. Kappa measures the agreement between the model’s predictions and the ground truth, while accounting for agreement occurring by chance, thus providing a more objective evaluation of classification performance. MCC evaluates the overall performance of a classification model, particularly suitable for handling imbalanced class distributions. The mathematical formulations of Accuracy, Precision, Recall, F1-Score, Kappa, and MCC are defined as follows (see Equations 1–6).


(1) $$\text{Accuracy}=\frac{TP+TN}{TP+TN+FP+FN}\times 100\%$$

(2) $$\text{Precision}=\frac{TP}{TP+FP}\times 100\%$$

(3) $$\text{Recall}=\frac{TP}{TP+FN}\times 100\%$$

(4) $$\text{F}1=\frac{2\times \text{Precision}\times \text{Recall}}{\text{Precision}+\text{Recall}}\times 100\%$$

(5) $$\text{Kappa}=\frac{P_{0}-P_{e}}{1-P_{e}}\times 100\%$$

(6) $$\text{MCC}=\frac{TP\times TN-FP\times FN}{\sqrt{\left(TP+FP)(TP+FN)(TN+FP)(TN+FN\right)}}\times 100\%$$

TP means the model correctly predicts a positive sample. TN means the model correctly predicts a negative sample. FP means the model wrongly predicts a negative sample as positive. FN means the model wrongly predicts a positive sample as negative. *p*_0_ represents the observed proportion of agreement, i.e., the percentage of instances where the raters reach consensus across all samples. *p_e_* represents the expected agreement by chance, assuming that the raters classify independently and randomly. In multi-class classification, macro and micro averages are common for evaluation. This study adopts macro average to compute Recall and Precision, and uses the global average value for Accuracy. For example, the macro-averaged Recall is calculated as shown in Equation 7.


(7) $$\text{Macro}\;\text{Recall}\;=\;\frac{1}{n}\;\sum_{i=1}^{n}{\text{Recall}}_{i}$$

### 4.3 Ablation study on SCEBD

To validate the effectiveness of the proposed method, ablation experiments are conducted on the SCEBD dataset. LCAMNet is an improved architecture based on the ShuffleNetV2 model (Zhang et al., 2018). The overall network structure is shown in **Table 3**.

**Table 3:** LCAMNet architecture diagram.

Layer	Output size	Attention	Ksize	Stride	Repeat	Output channels
Image	224x224					3
Conv1	112x112		3x3	2	1	24
MaxPool	56x56		3x3	2	1	24
stage2	28x28 28x28			2 1	1 1	48 48
Stage3	14x14 14x14			2 1	1 1	96 96
Stage4	7x7 7x7 7x7	1		2 1	1 1 1	192 192 192
Conv5	7x7		1x1	1	1	1024
GlobalPool	1x1		7x7			
FC						8

To verify the effectiveness of each proposed module, multiple comparative models are designed for experimentation. The baseline model is ShuffleNetV2. Model1 modifies only the downsampling module (SDSM) of ShuffleNetV2. Model2 modifies only the feature extraction module (SFEM). Model3 modifies both the feature extraction and downsampling modules based on ShuffleNetV2. Model4 further incorporates the improved triplet attention mechanism on top of Model3. The detailed configurations of the networks using different strategies are shown in **Table 4**.

**Table 4:** Network configurations with different strategies.

Model	Feature extraction	Downsampling	Attention
1	SFEM	DBDM	\
2	MFEM	SDSM	\
3	MFEM	DBDM	\
4	MFEM	DBDM	Improved triplet attention

The experimental results are shown in **Table 5**. GFLOPs measures the floating-point operations (in billions) and is used to evaluate the model’s computational complexity. Parameters refer to the number of trainable weights in the model and are commonly used to assess the model’s size. As seen in **Table 5**, Model1 outperforms the baseline, indicating that applying DBDM in the downsampling process of apple leaf disease images better preserves feature information compared to the original SDSM. Model2 also shows better performance than the baseline on the test set, suggesting that MFEM is more effective in extracting features from apple leaf disease images in natural environmental backgrounds than SFEM. Model3 performs better than both Model1 and Model2 on the test set, demonstrating that the combined application of MFEM and DBDM has a synergistic enhancement effect on apple leaf disease image classification, surpassing the performance of each method individually. Model4 shows further improvement over Model3. The addition of the enhanced triplet attention mechanism slightly increases the number of parameters but leads to a significant boost in performance. Additionally, compared to ShuffleNetV2, Model4 reduces both floating-point operations and parameter count while achieving a larger improvement in classification performance. In conclusion, through the stepwise introduction of DBDM, MFEM, and the improved triplet attention mechanism, the classification performance of the model continues to improve, validating the effectiveness of the proposed method.

**Table 5:** Ablation study on the SCEBD.

Models	Accuracy (%)	Recall (%)	Precision (%)	F1 (%)	Kappa (%)	MCC (%)	GFLOPs (G)	Parameters (M)
Baseline	88.01	88.00	88.98	88.21	86.30	86.48	0.04	1.31
Model1	88.78	88.80	89.46	88.97	87.17	87.24	0.03	1.29
Model2	91.33	91.36	91.83	91.43	90.09	90.13	0.03	1.28
Model3	92.35	92.34	92.73	92.41	91.25	91.28	0.03	1.29
Model4	92.60	92.64	92.85	92.64	91.55	91.58	0.03	1.30

**Figure 9:**
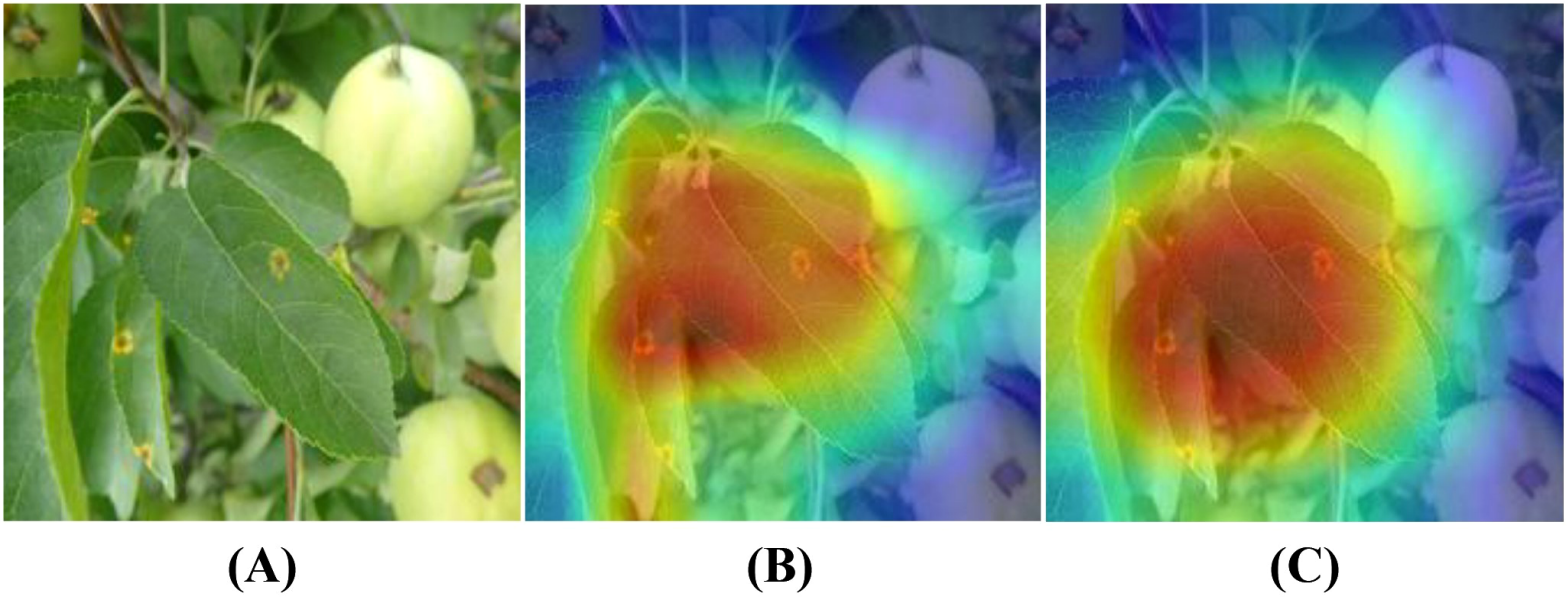
Grad-CAM visualization results of different models: **(A)** Original image; **(B)** Heatmap generated by the Baseline model; **(C)** Heatmap generated by Model4.

To further validate the effectiveness of the LCAMNet model in disease region identification, this study employs the Grad-CAM (Selvaraju et al., 2017) technique to visualize and analyze the model’s prediction results, comparing the performance before and after the improvements, as shown in **Figure 9**. **Figure 9A** presents the original image, **Figure 9B** shows the visualization result of the baseline model, and **Figure 9C** shows the visualization result of the LCAMNet model. Taking a rust disease image as an example, the baseline model focuses only on partial features of the diseased area, leading to missed and false detections, and fails to fully capture the lesion regions. In contrast, the LCAMNet model accurately localizes the key diseased regions of the apple leaf, with more comprehensive and discriminative feature extraction. These results demonstrate that the integration of the MFEM module, DBDM module, and the improved triplet attention mechanism in LCAMNet significantly enhances the model’s ability to focus on diseased areas, thereby improving its feature learning capacity and classification accuracy.

### 4.4 Performance comparison with classic convolutional neural networks on SCEBD

In this experiment, we compare LCAMNet with several classic convolutional neural network models, including VGG (Simonyan and Zisserman, 2014), ResNet (He et al., 2016), ResNext (Xie et al., 2017), and DenseNet (Huang et al., 2017), on SCEBD. The experimental results are shown in **Table 6**. These models exhibit issues when applied to disease classification tasks in natural environments, which are manifested in the following aspects: First, while VGG network has a simple structure, it suffers from a large number of parameters due to its deep architecture, making it prone to overfitting. As a result, its performance is relatively poor in the scenarios with small datasets or complex backgrounds. Second, ResNet and ResNext use residual connections to address the vanishing gradient problem. However, due to the deep network layers, they still struggle to effectively capture features in the presence of high noise and irregular lesions. DenseNet, despite having advantages in feature propagation, has a dense connectivity structure that leads to a large number of parameters and computational overhead, making it inefficient when processing high-resolution images. Thus, these four networks suffer from low computational efficiency and high model complexity.

**Table 6:** Performance comparison of LCAMNet and several classical CNN models on the SCEBD.

Models	Accuracy (%)	Recall (%)	Precision (%)	F1 (%)	Kappa (%)	MCC (%)	GFLOPs (G)	Parameters (M)
VGG13	89.03	89.04	89.15	89.03	87.64	87.53	11.36	133.05
ResNet34	88.52	88.54	89.02	88.66	86.88	86.94	3.68	21.80
ResNext50	85.97	85.99	86.48	86.12	83.96	84.00	4.29	25.03
DenseNet201	90.56	90.56	90.67	90.60	89.21	89.24	4.39	20.01
GoogLeNet	92.09	92.09	92.20	92.11	90.96	90.98	1.60	7.01
InceptionRes-NetV2	91.58	91.59	92.14	91.71	90.38	90.39	6.50	55.84
EfficientNet-B0	91.33	91.35	91.54	91.34	90.09	90.13	0.42	8.43
ShuffleNetV2	88.01	88.00	88.98	88.21	86.30	86.48	0.04	1.31
MobileNetV2	89.80	89.79	90.52	89.93	88.34	88.41	0.33	3.51
GhostNet	90.31	90.35	91.09	90.45	88.92	88.98	0.16	5.18
LCAMNet	**92.60**	**92.64**	**92.85**	**92.64**	**91.55**	**91.58**	**0.03**	**1.30**

Bold values indicate the best results in each column. For Accuracy, Recall, Precision, F1, Kappa, and Matthews Correlation Coefficient (MCC), higher values indicate better performance. Conversely, for GFLOPs and Parameters, lower values indicate a more lightweight and efficient model.

GoogLeNet (Szegedy et al., 2015) designs the Inception module, which applies multiple convolutional kernels of different sizes in parallel to capture features at various scales. This improves both feature representation and computational performance. Although InceptionResNetV2 (Szegedy et al., 2016) incorporates optimization techniques such as multi-branch convolutions, residual connections, and depthwise separable convolutions to improve performance, the network’s complexity and large number of parameters limit its advantages in some application scenarios.

EfficientNetB0 (Tan and Le, 2020), ShuffleNetV2 (Zhang et al., 2018), MobileNetV2 (Howard et al., 2017), and GhostNet (Han et al., 2020) are lightweight models that have been widely used in recent years. These models optimize the network structure to reduce computational overhead while maintaining high accuracy. They incorporate various lightweight techniques, such as depthwise separable convolutions, channel reparameterization, and residual connections, achieving a good balance between computational efficiency and model accuracy. However, these models may face performance bottlenecks when handling more complex tasks due to limited model capacity and expressive power.

**Figure 10:**
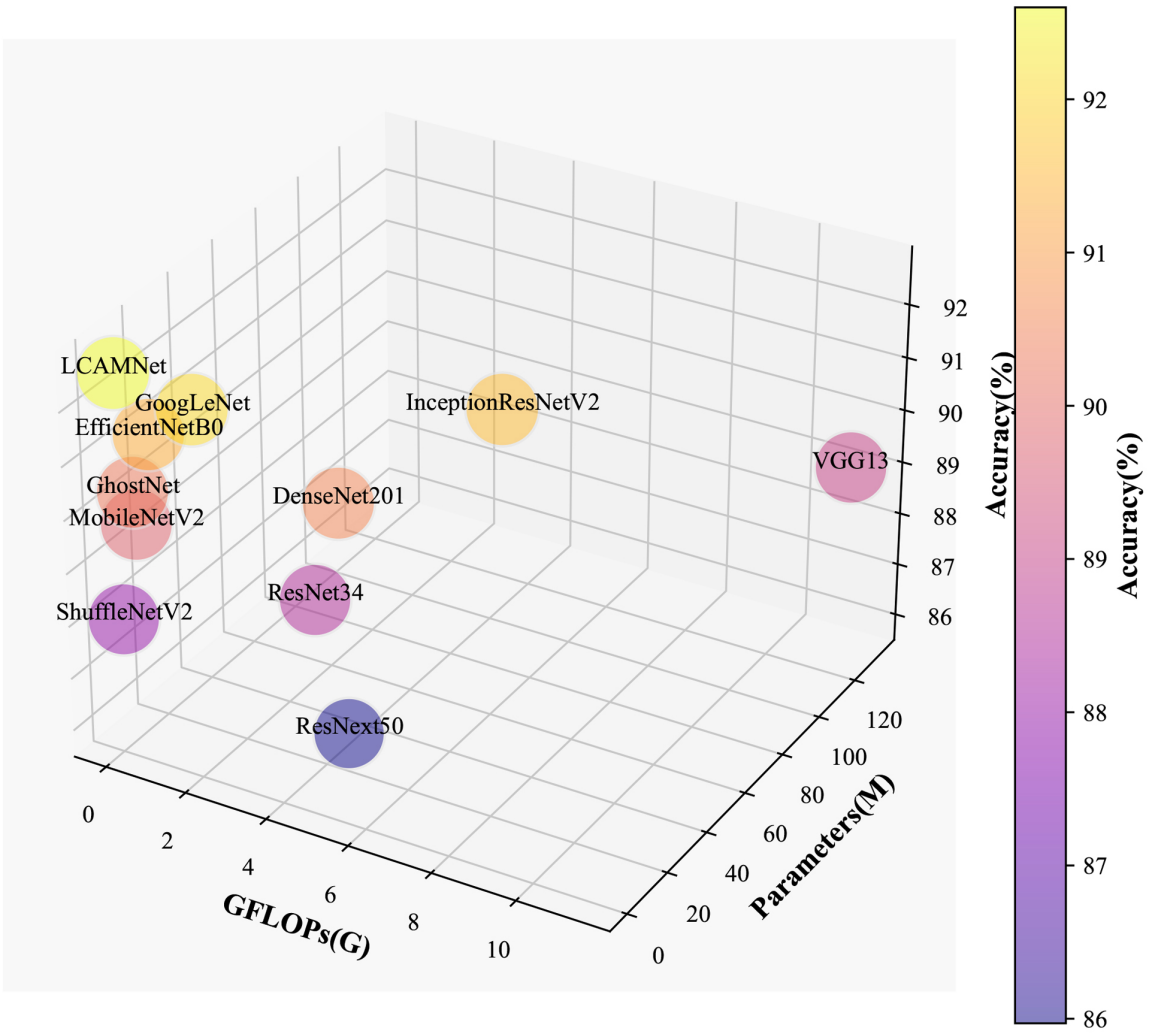
3D Visualization of accuracy, GFLOPs, and parameter count on the SCEBD.

LCAMNet, based on ShuffleNetV2 model, optimizes the feature extraction and downsampling modules. By using depthwise separable convolutions and structural reparameterization, it reduces network parameters while still extracting sufficient features. Compared to classic convolutional neural networks, LCAMNet has fewer parameters than ShuffleNetV2 and achieves the highest accuracy. This demonstrates that LCAMNet is a high-performance, lightweight model for apple leaf disease classification. The performance comparison of LCAMNet and several classical CNN models on SCEBD is shown in **Table 6**. The three-dimensional visual analysis of accuracy, GFLOPs, and parameter count on SCEBD is presented in **Figure 10**.

### 4.5 Performance comparison with similar crop disease image classification models on SCEBD

This study compares the performance of LCAMNet with similar crop disease image classification models on the SCEBD. Re-GoogLeNet (Yang et al., 2023) is a network designed for rice image classification in natural environmental backgrounds, based on a series of improvements to GoogLeNet. First, the 7×7 convolution kernel in the first layer of GoogLeNet is replaced with three consecutive 3×3 convolutions. Then, the Inception module is enhanced by adding the ECA attention mechanism and optimized residual connections to strengthen information flow. Lastly, LeakyReLU replaces the ReLU activation to better capture irregular features in diseased leaves. ALS-Net (Liu et al., 2022a) adds an Inception structure after 3×3 convolution in the ShuffleNetV2 model for multi-scale feature extraction. Additionally, the 3×3 convolutions in the ShuffleNet block are replaced with 5×5 depthwise convolutions to obtain the ALS module. The ELU activation function also replaces ReLU, addressing gradient vanishing and neuron death issues. To further improve classification performance, ALS-Net uses DenseNet161 as a teacher network to guide training and enhance the model’s classification ability. LBMRNet (Li et al., 2023) is a lightweight algorithm for recognizing tomato leaf diseases, aimed at tackling the problem of significant variation within the same class and minimal variation between different classes in tomato leaf disease images. LBMRNet consists of alternating complementary group dilation residual (CGDR) modules and visual enhancement modules. The CGDR module uses a multi-branch design to capture various features of tomato leaf diseases from different receptive fields. It incorporates multiple residual connections to facilitate better information flow across the network layers. The visual enhancement module combines average pooling, max pooling, and 1×1 convolutions as downsampling strategies, effectively fusing the visual enhancement effects and preventing information loss during the downsampling process, thus improving classification accuracy.

**Figure 11:**
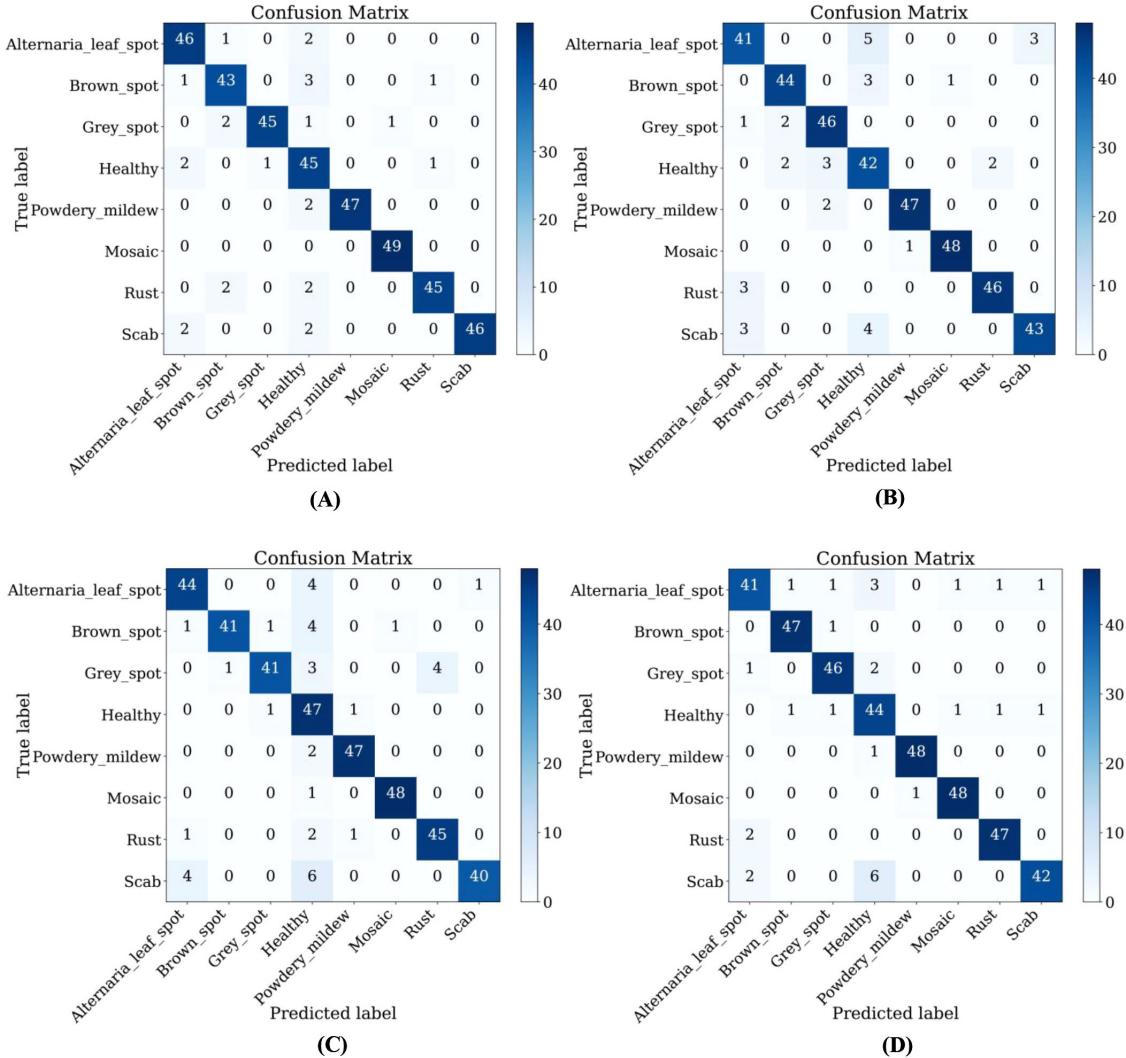
Confusion matrices of LCAMNet and similar crop disease image classification models on SCEBD: **(A)** Re-GoogLeNet; **(B)** ALS-Net; **(C)** LBMRNet; **(D)** LCAMNet.

From **Table 7**, it could be seen that the performance of ALS-Net and LBMRNet are inferior to LCAMNet (for LBMRNet, only the network structure is restored). This is likely because, although ALS-Net improves ShuffleNetV2, the multi-scale feature extraction is only performed at the initial stages of the network and fails to effectively integrate these features at each stage, limiting its feature extraction capability. Although LBMRNet improves the feature extraction and downsampling layers, its multi-residual structure leads to the continuous retention of irrelevant information, affecting the recognition performance. In comparison, Re-GoogLeNet optimizes GoogLeNet by introducing the attention mechanism to preserve important features and uses residual connections to retain original features. While its performance is better than that of LCAMNet, its higher FLOPs and Parameters may limit its applicability in resource-constrained scenarios. The confusion matrices of the models are shown in **Figure 11**.

**Table 7:** Performance comparison of LCAMNet and similar crop disease image classification models on the SCEBD.

Models	Accuracy (%)	Recall (%)	Precision (%)	F1 (%)	Kappa (%)	MCC (%)	GFLOPs (G)	Parameters (M)
Re-GoogLeNet	**93.37**	**93.36**	**93.79**	**93.46**	**92.42**	**92.64**	2.80	9.11
ALS-Net	91.07	91.09	91.28	91.13	89.80	89.81	0.73	1.18
LBMRNet	90.05	90.06	91.54	90.30	88.63	88.81	0.17	**0.91**
LCAMNet	92.60	92.64	92.85	92.64	91.55	91.58	**0.03**	1.30

Bold values indicate the best results in each column. For Accuracy, Recall, Precision, F1, Kappa, and Matthews Correlation Coefficient (MCC), higher values indicate better performance. Conversely, for GFLOPs and Parameters, lower values indicate a more lightweight and efficient model.

### 4.6 Performance comparison on the public dataset FGVC8

**Figure 12:**
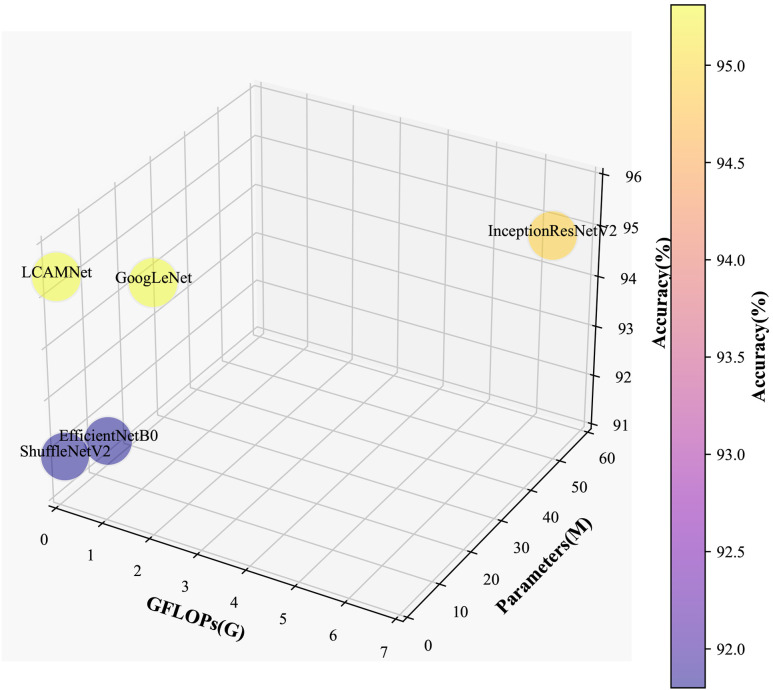
3D visualization of accuracy, FLOPs, and parameters on the FGVC8 dataset.

This study compares the performance of LCAMNet with several classical CNN models on the FGVC8 dataset to verify its effectiveness and superiority. Detailed experimental settings are provided in Section 4.1. **Table 8** presents a performance comparison between LCAMNet and several classical CNN models on FGVC8. The results show that LCAMNet achieves a classification accuracy comparable to GoogLeNet and InceptionResNetV2, while significantly reducing FLOPs and Parameters compared to these two models. This indicates that LCAMNet maintains a high recognition accuracy while lowering computational resource consumption. In addition, LCAMNet achieves better classification accuracy than ShuffleNetV2 on the public dataset, while maintaining slightly lower FLOPs and parameters. This demonstrates that LCAMNet effectively integrates techniques like multi-branch feature extraction modules, dual-branch downsampling modules, and attention mechanisms into the ShuffleNetV2 architecture, making it suitable for different datasets. Three-dimensional visual analysis of accuracy, GFLOPs, and parameters on FGVC8 is presented in **Figure 12**.

**Table 8:** Performance comparison of LCAMNet and several classical CNN models on the FGVC8 dataset.

Models	Accuracy (%)	Recall (%)	Precision (%)	F1 (%)	Kappa (%)	MCC (%)	GFLOPs (G)	Parameters (M)
GoogLeNet	**95.31**	**95.33**	95.56	**95.39**	94.20	95.39	1.60	7.01
InceptionRes-NetV2	94.92	94.93	95.40	95.06	93.71	95.06	6.50	55.84
EfficientNet-B0	91.80	91.88	92.13	91.92	89.76	91.92	0.42	8.43
ShuffleNetV2	91.80	91.96	91.89	91.86	89.91	91.86	0.04	1.31
LCAMNet	**95.31**	95.29	**95.81**	**95.39**	**94.22**	**95.39**	**0.03**	**1.30**

Bold values indicate the best results in each column. For Accuracy, Recall, Precision, F1, Kappa, and Matthews Correlation Coefficient (MCC), higher values indicate better performance. Conversely, for GFLOPs and Parameters, lower values indicate a more lightweight and efficient model.

**Figure 13:**
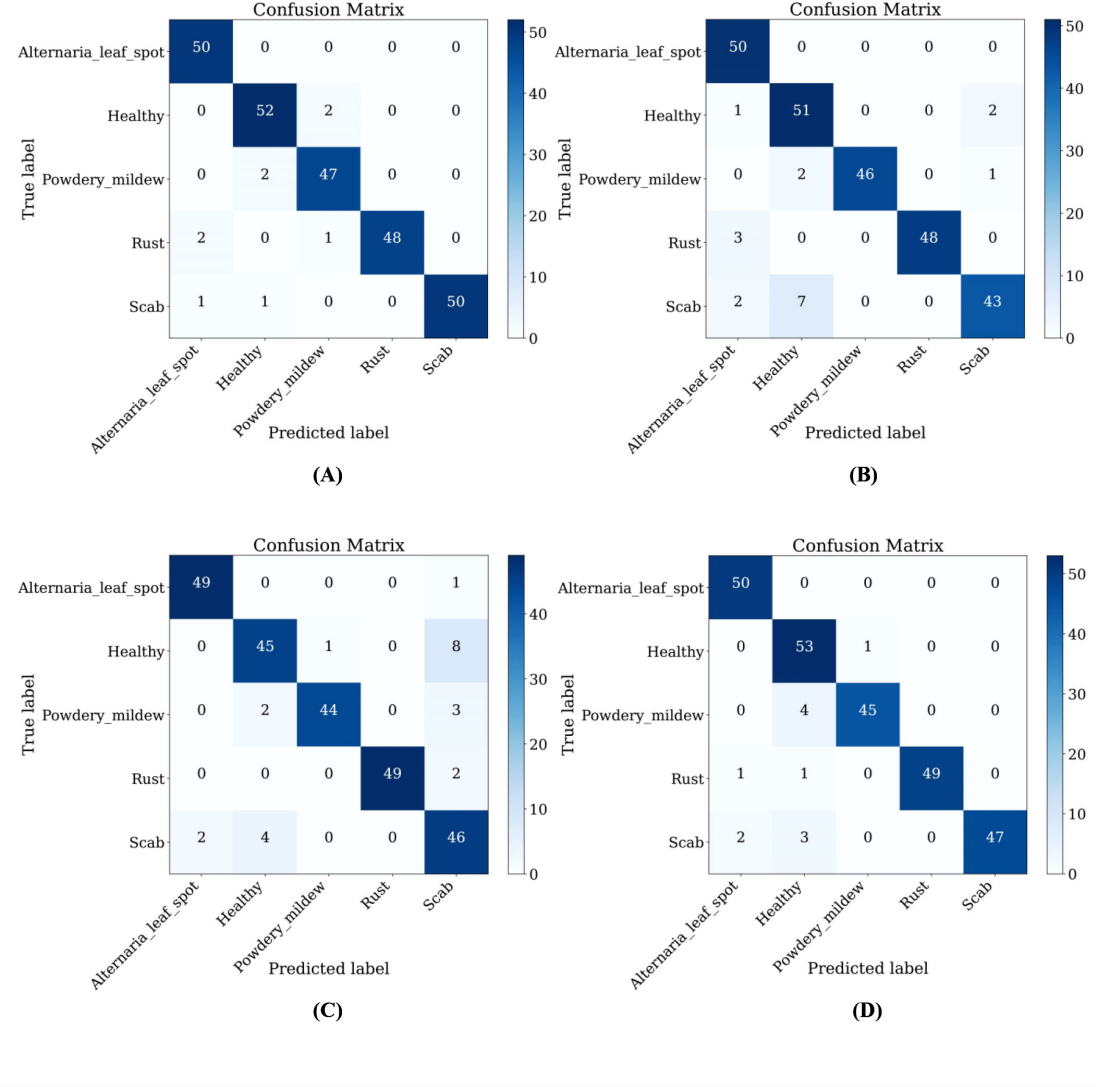
Confusion matrices of similar crop disease image classification models on the FGVC8 dataset: **(A)** Re-GoogLeNet; **(B)** ALS-Net; **(C)** LBMRNet; **(D)** LCAMNet.

Furthermore, LCAMNet is compared with similar crop disease image classification models. Experimental results show that LCAMNet outperforms ALS-Net and LBMRNet in overall performance. Although its performance is slightly lower than Re-GoogLeNet, LCAMNet has significantly fewer FLOPs and Parameters, demonstrating a notable optimization in computational resource consumption. In conclusion, the experimental results fully validate the effectiveness of LCAMNet on the public dataset. LCAMNet achieves competitive recognition accuracy while significantly reducing computational complexity, showcasing strong practical application value and potential for broader deployment. Detailed results can be found in **Table 9**, and the models’ confusion matrices are shown in **Figure 13**.

**Table 9:** Performance comparison of similar crop disease image classification models on the FGVC8 dataset.

Models	Accuracy (%)	Recall (%)	Precision (%)	F1 (%)	Kappa (%)	MCC (%)	GFLOPs (G)	Parameters (M)
Re-GoogLeNet	**96.48**	**96.50**	**96.58**	**96.49**	**95.60**	**96.63**	2.80	9.11
ALS-Net	92.97	93.03	93.55	93.08	91.21	91.32	0.73	1.18
LBMRNet	91.02	91.13	91.75	91.30	88.77	88.84	0.17	**0.91**
LCAMNet	95.31	95.29	95.81	95.39	94.14	94. 22	**0.03**	1.30

Bold values indicate the best results in each column. For Accuracy, Recall, Precision, F1, Kappa, and Matthews Correlation Coefficient (MCC), higher values indicate better performance. Conversely, for GFLOPs and Parameters, lower values indicate a more lightweight and efficient model.

### 4.7 Model limitation analysis

Although LCAMNet achieves outstanding performance on both the SCEBD and FGVC8 datasets, several limitations remain based on the experimental results:

As shown in **Table 6**, LCAMNet demonstrates a favorable balance between accuracy and model complexity compared to various CNN models on the SCEBD dataset. However, in the comparative experiments with other crop disease identification models **Table 7**, although LCAMNet outperforms ALS-Net and LBMRNet, its accuracy is slightly lower than that of Re-GoogLeNet. This indicates that while LCAMNet holds significant advantages in terms of parameter count and computational cost, there is still room for improvement in feature fusion and deep representation capabilities. Moreover, the staged multi-scale attention mechanism present in Re-GoogLeNet significantly enhances performance, suggesting that LCAMNet could benefit from incorporating richer stage-wise fusion strategies for further optimization.

Furthermore, LCAMNet maintains competitive performance on the FGVC8 dataset (as shown in **Tables 8**, **9**), indicating its generalization capability. However, some classification confusion still occurs among certain categories in FGVC8, as evidenced by the confusion matrix in **Figure 13**. This reveals a risk of misclassification, especially when dealing with disease symptoms that have similar morphological patterns and subtle color differences. These observations suggest that LCAMNet could further enhance its fine-grained modeling capacity for complex lesion patterns.

## 5 Conclusion

The lightweight converged attention multi-branch network, LCAMNet, proposed in this study achieves high accuracy in apple leaf disease classification while significantly reducing model complexity and computational cost. This demonstrates its strong potential for deployment in resource-constrained and complex natural environments. By integrating structural re-parameterization, dual-branch downsampling, and multi-scale attention mechanisms, LCAMNet effectively enhances lesion feature modeling and the expression of feature diversity.

Despite its outstanding performance in apple leaf disease recognition, there remains room for improvement in LCAMNet’s adaptability and generalization capability. Future research will focus on two main directions: (1) systematically evaluating the model’s robustness and stability under complex natural conditions, such as varying illumination, camera angles, and degrees of leaf occlusion; and (2) extending the application of LCAMNet to the classification of diseases in other crops such as wheat, maize, and rice, in order to explore its cross-crop generalization performance and domain adaptability. These efforts will further promote the practical deployment of LCAMNet in multi-scenario and multi-crop disease recognition, laying a solid foundation for building an intelligent plant disease identification system in precision agriculture.

## Data Availability

Publicly available datasets were analyzed in this study. This data can be found here: https://www.kaggle.com/competitions/plant-pathology-2021-fgvc8; https://github.com/JasonYangCode/AppleLeaf9; https://www.scidb.cn/en/detail?dataSetId=0e1f57004db842f99668d82183afd578.
